# Formation of *a*-plane facets in three-dimensional hexagonal GaN structures for photonic devices

**DOI:** 10.1038/s41598-017-09782-1

**Published:** 2017-08-24

**Authors:** Seung-Hyuk Lim, Young Chul Sim, Yang-Seok Yoo, Sunghan Choi, Sangwon Lee, Yong-Hoon Cho

**Affiliations:** 1 Department of Physics, Korea Advanced Institute of Science and Technology, Daejeon, 34141 Republic of Korea; 2 Department of Physics, Present Address: Chemistry, and Biology (IFM), Semiconductor Materials, Linköping University, SE-58183 Linköping, Sweden

## Abstract

Control of the growth front in three-dimensional (3D) hexagonal GaN core structures is crucial for increased performance of light-emitting diodes (LEDs), and other photonic devices. This is due to the fact that InGaN layers formed on different growth facets in 3D structures exhibit various band gaps which originate from differences in the indium-incorporation efficiency, internal polarization, and growth rate. Here, *a*-plane {${\bf{11}}\bar{{\bf{2}}}{\bf{0}}$ } facets, which are rarely formed in hexagonal pyramid based growth, are intentionally fabricated using mask patterns and adjustment of the core growth conditions. Moreover, the growth area covered by these facets is modified by changing the growth time. The origin of the formation of *a*-plane {${\bf{11}}\bar{{\bf{2}}}{\bf{0}}$} facets is also discussed. Furthermore, due to a growth condition transition from a 3D core structure to an InGaN multi-quantum well, a growth front transformation (i.e., a transformation of *a*-plane {${\bf{11}}\bar{{\bf{2}}}{\bf{0}}$} facets to semi-polar {${\bf{11}}\bar{{\bf{2}}}{\bf{2}}$} facets) is directly observed. Based on our understanding and control of this novel growth mechanism, we can achieve efficient broadband LEDs or photovoltaic cells.

## Introduction

Established techniques for fabricating group III-nitride semiconductor have resulted in many applications suited for commercial devices, such as light-emitting diodes (LEDs), laser diodes, photodetectors, photovoltaic devices, and radio frequency power devices^1–3^. However, the presence of large piezoelectric fields (~MV/cm) along the *c*-axis in these materials reduces the efficiency of these devices. In order to improve the performance of such devices, nitride heterostructures and quantum wells (QWs) need to be grown along specific crystallographic directions where the piezoelectric field is negligible. Up to now, semi-polar and non-polar GaN facets, including *a*-polar {$11\bar{2}0$} facets grown on an *r*-plane {$01\bar{1}2$} sapphire substrate by metal-organic chemical vapor deposition (MOCVD), have been studied by several groups^4–9^. Two common approaches exist to obtain semi-polar and/or non-polar plane facets. In the first case, we can obtain either semi-polar or non-polar GaN planes using some substrates with specific orientations, such as {001} 7°-off silicon, {113} silicon, *r*-plane {$01\bar{1}2$} sapphire, and *n*-plane {$11\bar{2}6$} sapphire substrates^10^. The second method used to obtain semi-polar and/or non-polar GaN planes is by making three-dimensional (3D) structures using selective area growth (SAG) techniques^8, 11–14^. B. Leung *et al*. have recently suggested that the determination and utilization of kinetic Wulff plots (i.e., *v*-plots) under various MOCVD growth conditions, offers a cohesive and rational model for GaN heteroepitaxy along polar, non-polar, and semi-polar orientations^9^. Since then, we have recently reported on the fabrication of double concentric truncated pyramid structures^13^ that contain not only polar (0001), semi-polar {$10\bar{1}1$}, and {$11\bar{2}2$} facets, but also non-polar *a*-plane {$11\bar{2}0$} facets which are rarely obtained by conventional 3D SAG growth, and which contribute to a high color rendering index in white LEDs. Although many pioneering growth mechanisms of *m*-plane {$10\bar{1}0$} facets have published^15–19^ and {$11\bar{2}0$} facets are studied in case of stripe based structures^20, 21^, the origin of how {$11\bar{2}0$} facets are formed in hexagonal structures still remains unanswered. Indeed, if we could understand and control the growth facets of a 3D GaN core structure, various band-gap energies of the InGaN layer could be achieved due to differences in the indium-incorporation efficiency, internal polarization, and growth rate, which could then be utilized in advanced broadband emitters, or photovoltaic cells.

In this work, control of the growth front area in SAG 3D GaN structures with various crystal facets is investigated. In addition, we discuss the origin of unusual *a*-plane {$11\bar{2}0$} crystal facets. The formation of the *a*-plane facets is explained by the geometric circular patterning used, with the area covered by growth facets capable of being adjusted by the variation of growth time. Finally, we also directly observe the gradual transformation of a growth front at the interface between the 3D core structure and a QW layer, which leads to various interesting characteristics within a single 3D structure.

## Results and Discussion

### Fabrication and overview of 3D SAG structures

**Figure 1:**
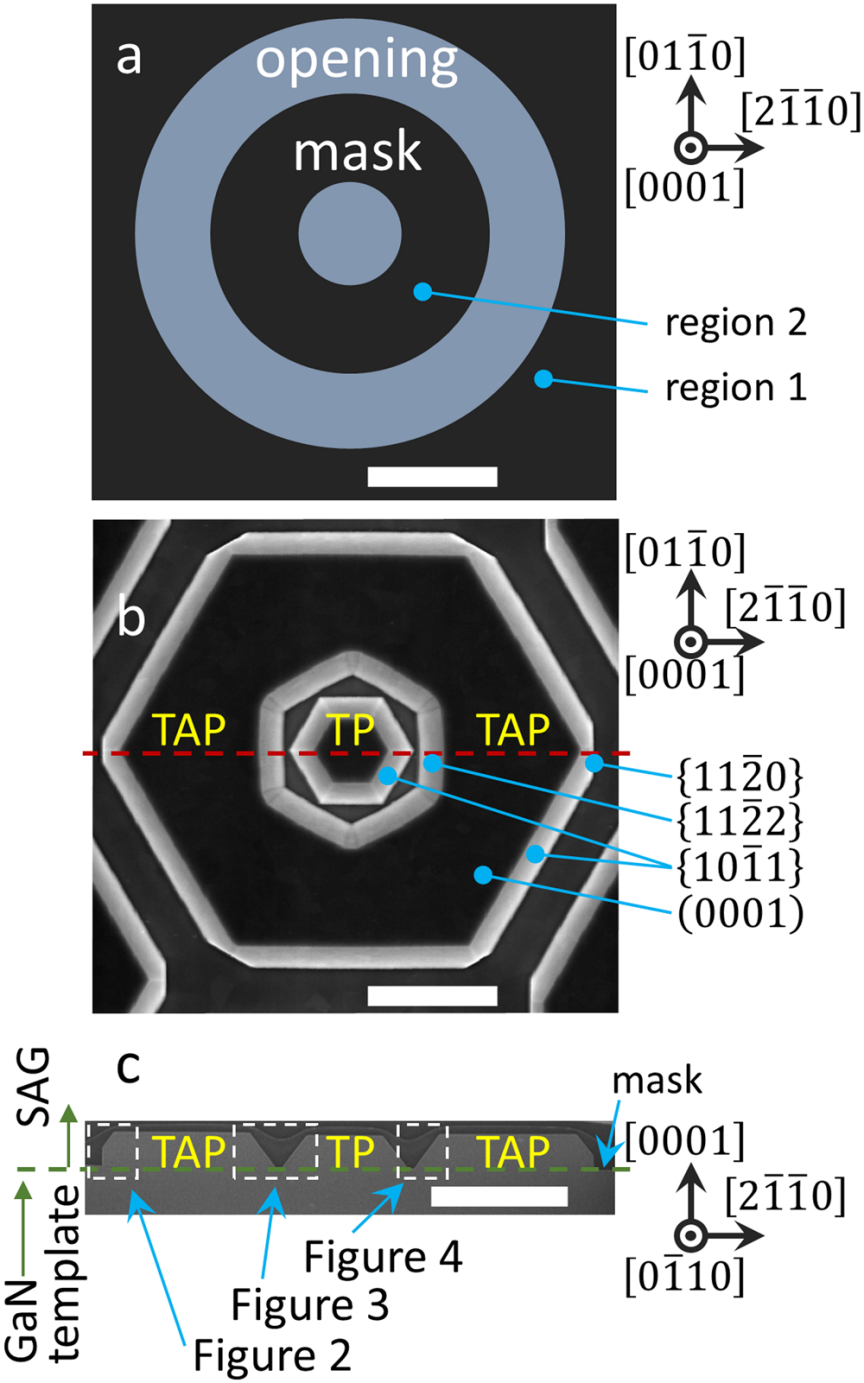
(**a**) A top-down view schematic of the dielectric mask used in our SAG process. (**b**) Overhead, and (**c**) cross-sectional SEM images of sample B after *p*-GaN layer growth. (Scale bars: 5 μm).

Our 3-μm-thick GaN templates were prepared by MOCVD. After a SiN_*x*_ dielectric mask was deposited on the template, concentric circle and annular openings were then cut from the template using ultraviolet lithography and reactive-ion etching techniques, as shown in Fig. 1(a). The opening diameters of the circle, inner ring, and outer ring were 3, 9, and 15 μm, respectively. The template wafer was then loaded back into the MOCVD reactor, and an *n*-type GaN layer was epitaxially overgrown on the wafer for various growth times. In order to investigate the evolution of the growth front with growth time, we used the different SAG times of 900, 1200, and 1500 s for samples A, B, and C, respectively. For the growth of the *n*-GaN 3D core structure, the V/III ratio, growth pressure, and temperature were set as 60, 100 Torr, and 1040 °C, respectively. In the case of sample B, five pairs of InGaN/GaN multi-quantum wells (MQWs), *p*-AlGaN, and a *p*-GaN layer were sequentially fabricated on the wafer in order to elucidate the evolution of the growth front in 3D LED structures. The growth parameters of the MQWs, *p*-AlGaN, and *p*-GaN were different to those of the *n*-GaN 3D core structure, and are detailed in Table 1. From the table, it is seen that growth from MQWs to *p*-GaN has a lower growth temperature, but a much higher V/III ratio than those of *n*-GaN 3D core structures. Figure 1(b) shows a top-down view of a scanning electron microscopy (SEM) image of the grown sample. A truncated pyramid (TP), and truncated annular pyramid (TAP) structures are hexagonally fabricated on the circular and annular openings, respectively. Figure 1(c) displays a cross-sectional view of an SEM image after focused ion beam (FIB) milling along the red dashed line marked in Fig. 1(b) (i.e., [$2\bar{1}\bar{1}0$]). The green horizontal dashed line in Fig. 1(c) indicates the mask height at which the SAG begins. The semi-polar {$10\bar{1}1$} facets and *c*-plane (0001) facets are formed in common with TP and TAP structures. However, additional inner semi-polar {$11\bar{2}2$} facets, and outer *a*-plane {$11\bar{2}0$} facets are formed for the TAP structure. As B. Leung *et al*. discussed^9^, the growth front formation of inner semi-polar {$11\bar{2}2$} facets which are saddle points of the 3D *v*-plot can be explained by a concave growth mode.

**Table 1:** Growth parameters of 3D structure LEDs.

Layer	Growth temperature (°C)	Growth pressure (torr)	V/III ratio
*p*-GaN	970	100	133
*p*-AlGaN	970	100	133
Quantum wells	680	100	4000
Quantum barriers	850	100	1000
*n*-GaN 3D structure	1040	100	60

### Time evolution of growth front and cross-sectional characterization

**Figure 2:**
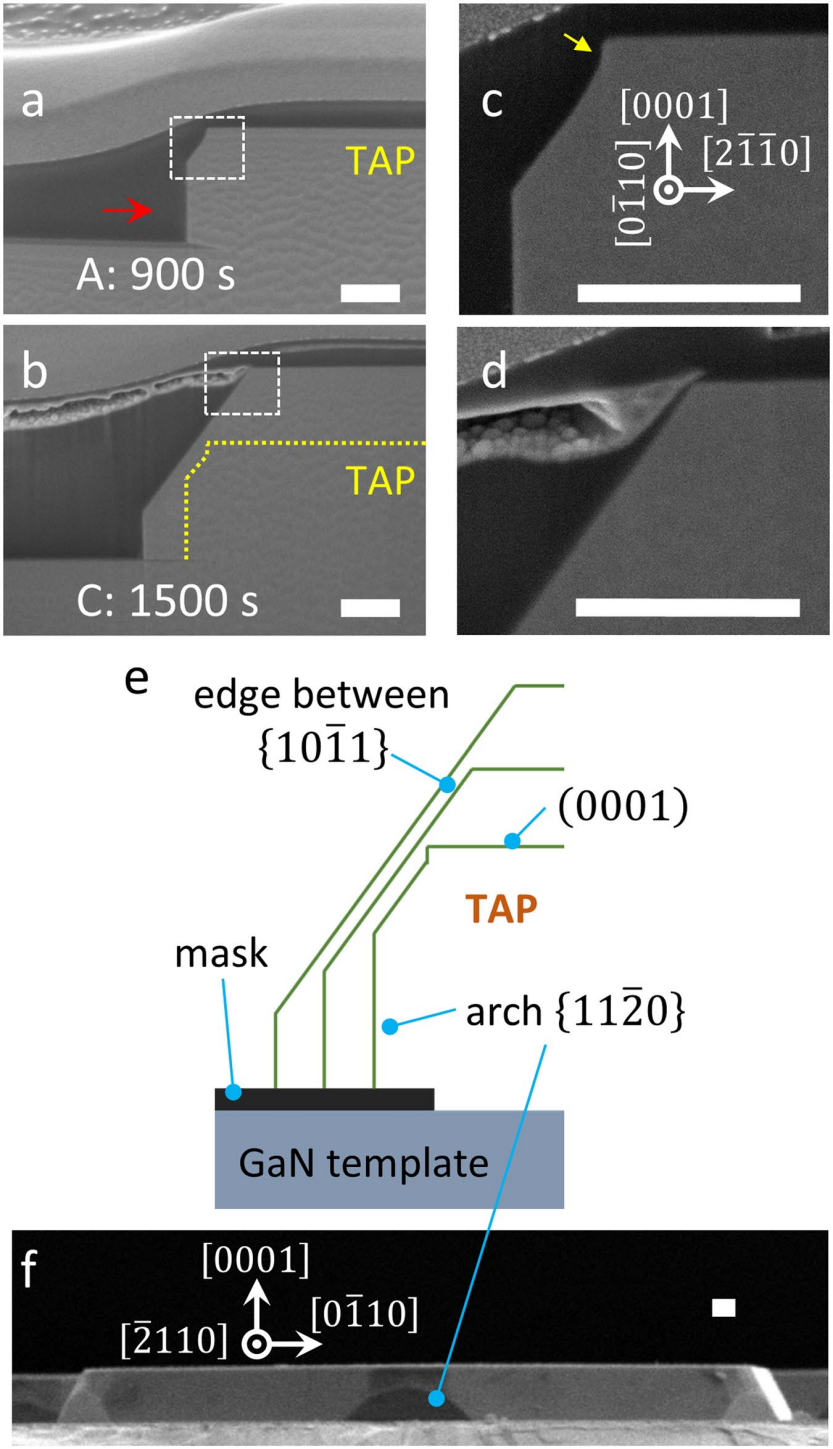
Cross-sectional view SEM images of outer *a*-plane {$11\bar{2}0$} facets of (**a**) sample A, and (**b**) sample C. (**c**) High magnification SEM images of sample A, and (**d**) sample C. The yellow guide line in (**b**) indicates the growth front of sample A. (**e**) Cross-sectional schematic representing the time evolution of outer *a*-plane {$11\bar{2}0$} facets. (**f**) Cross-sectional SEM image along the [$2\bar{1}\bar{1}0$] direction. (Scale bars: 0.5 μm).

In order to explain the formation of *a*-plane {$11\bar{2}0$} facets, magnified cross-sectional SEM images of samples A and C (white dashed box in Fig. 1(c)) were taken, as shown in Fig. 2. Due to the convex growth for the outer facet growth direction, the slowest semi-polar {$10\bar{1}1$} facets become dominant for the TP structure and exterior of the TAP structure. However, if the growth time is appropriately controlled, the growth outside of the TAP area can be in an intermediate state before the full semi-polar {$10\bar{1}1$} facets are formed. This means that the second slowest, arch-shaped, non-polar *a*-plane {$11\bar{2}0$} facets can be generated, which are indicated by the red arrow in Fig. 2(a). Figure 2(a–d) show cross-sectional SEM images of samples A and C, with Fig. 2(c,d) being higher magnification views of those shown in Fig. 2(a,b), respectively. A yellow guideline in Fig. 2(b) indicates the growth front of sample A (Fig. 2(a)). According to Fig. 2(a,b), the cross-sectional schematic of time evolution is illustrated in Fig. 2(e) based on real scale. As the growth time increases, the growth front area for the arch-shaped *a*-plane {$11\bar{2}0$} facets decrease. This result is significant since we could thus obtain and intentionally design structures with non-polar facets simply by adjusting the growth time. We note that we also observe *a*-plane {$11\bar{2}0$} facets (indicated by the yellow arrow in Fig. 2(c)) above the edge between semi-polar {$10\bar{1}1$} facets in the early growth stage of sample A. This formation may occur because adatoms prefer to be epitaxially grown on *a*-plane {$11\bar{2}0$} facets in early stages of growth. Though as time proceeds, the growth front is well organized by the semi-polar {$10\bar{1}1$} facets with slowest growth rate, as shown in Fig. 2(d). However, even after extended growth times, we can observe an arch shape along the [$2\bar{1}\bar{1}0$] direction, as shown in the SEM image of Fig. 2(f), where the direction of this facet corresponds to the direction indicated by the red arrow shown in Fig. 2(a).

**Figure 3:**
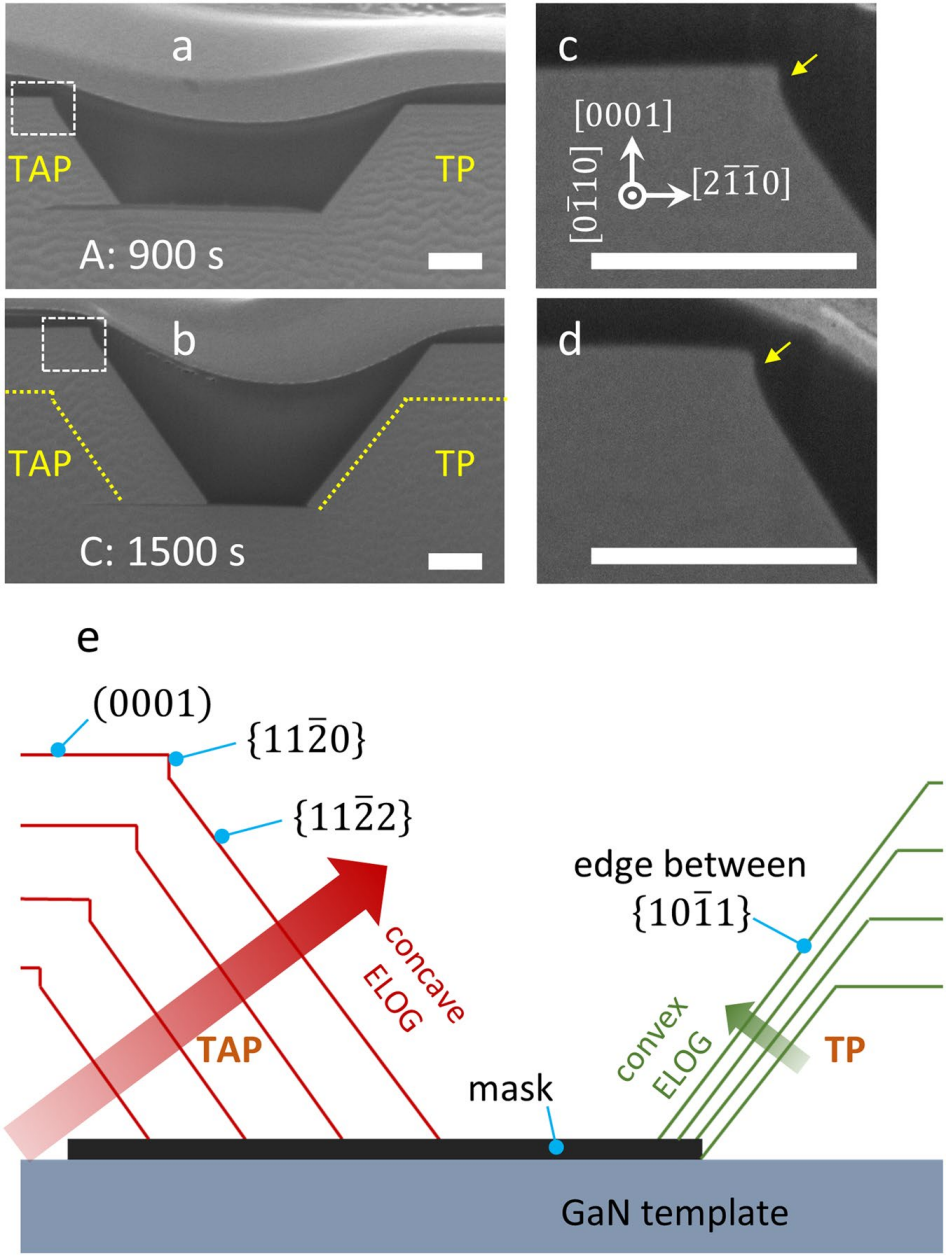
Cross-sectional view SEM images of inner TAP semi-polar {$11\bar{2}2$} facets and the TP edge between {$10\bar{1}1$} facets of (**a**) sample A, and (**b**) sample C. (**c**) High magnification SEM images of sample A, and (**d**) sample C. The yellow guideline in (**b**) indicates the growth front of sample A. (**e**) Cross-sectional schematic representing the time evolution of inner TAP semi-polar {$11\bar{2}2$}, and TP edge {$10\bar{1}1$} facets. (Scale bars: 0.5 μm).

Figure 3 displays SEM images and a schematic of the region between the TAP and TP structures along the same directions as those shown in Fig. 2. In Fig. 3(a,b), convex-shaped epitaxially lateral overgrowth (convex ELOG), and concave-shaped ELOG (concave ELOG) directions are seen simultaneously. Since we take the SEM image along the < $0\bar{1}10$ > axis, the distance of semi-polar {$10\bar{1}1$} convex ELOG is overestimated (i.e., the lateral growth distance: $(\{10\bar{1}1\}+r)=(\sqrt{3}/2)\times$ (edge between {$10\bar{1}1$} + *r*), where *r* is the radius of the circular opening). Further details regarding this point are provided in the Supplementary Information. As a result, the measured concave lateral growth rate is found to be over 13.4 times faster than the deduced convex lateral growth rate under our growth conditions. In case of concave ELOG, only semi-polar {$11\bar{2}2$} facets remain since they correspond to a solitary saddle point in the 3D *v*-plots for the Ga-polar planes^9^ (i.e., the northern hemisphere in 3D *v*-plots, 0 < *θ* < π/2, where *θ* is the polar angle in 3D *v*-plots). Since the growth rate of 3D structures is related to the migration of Ga adatoms^19, 22, 23^, the height of 3D structures depends on the surrounding mask area, when the length of the region covered by the mask is shorter than the migration length of the Ga adatoms. As a result, the height of the TP structure is slightly lower than that of the TAP structures, as shown in Fig. 3(b,e). This is because the TAP structure receives migratory adatoms from the mask outside (region 1, Fig. 1(a)), while the TP structure can only receive adatoms from the narrow region between the TAP and TP structures (region 2, Fig. 1(a)). We have also found *a*-plane {$11\bar{2}0$} facets (indicated by the yellow arrow in Fig. 3(c,d)) above the semi-polar {$11\bar{2}2$} facets. In contrast with the *a*-plane {$11\bar{2}0$} facets on the exterior of the TAP structure, which disappear after extended growth times (over 1500 s) (Fig. 2(c)), those *a*-plane facets found inside the TAP structure (Fig. 3(c)) remain, even for growth times greater than 1500 s. This phenomenon might be associated with the Ga-rich environment (i.e., low V/III ratio). Under Ga-rich environment, *a*-plane {$11\bar{2}0$} facets (non-polar) would be selected as a growth front than semi-polar {$11\bar{2}2$} facets (nitrogen-polarity)^11^.

### Observation and analysis of growth front transformation

**Figure 4:**
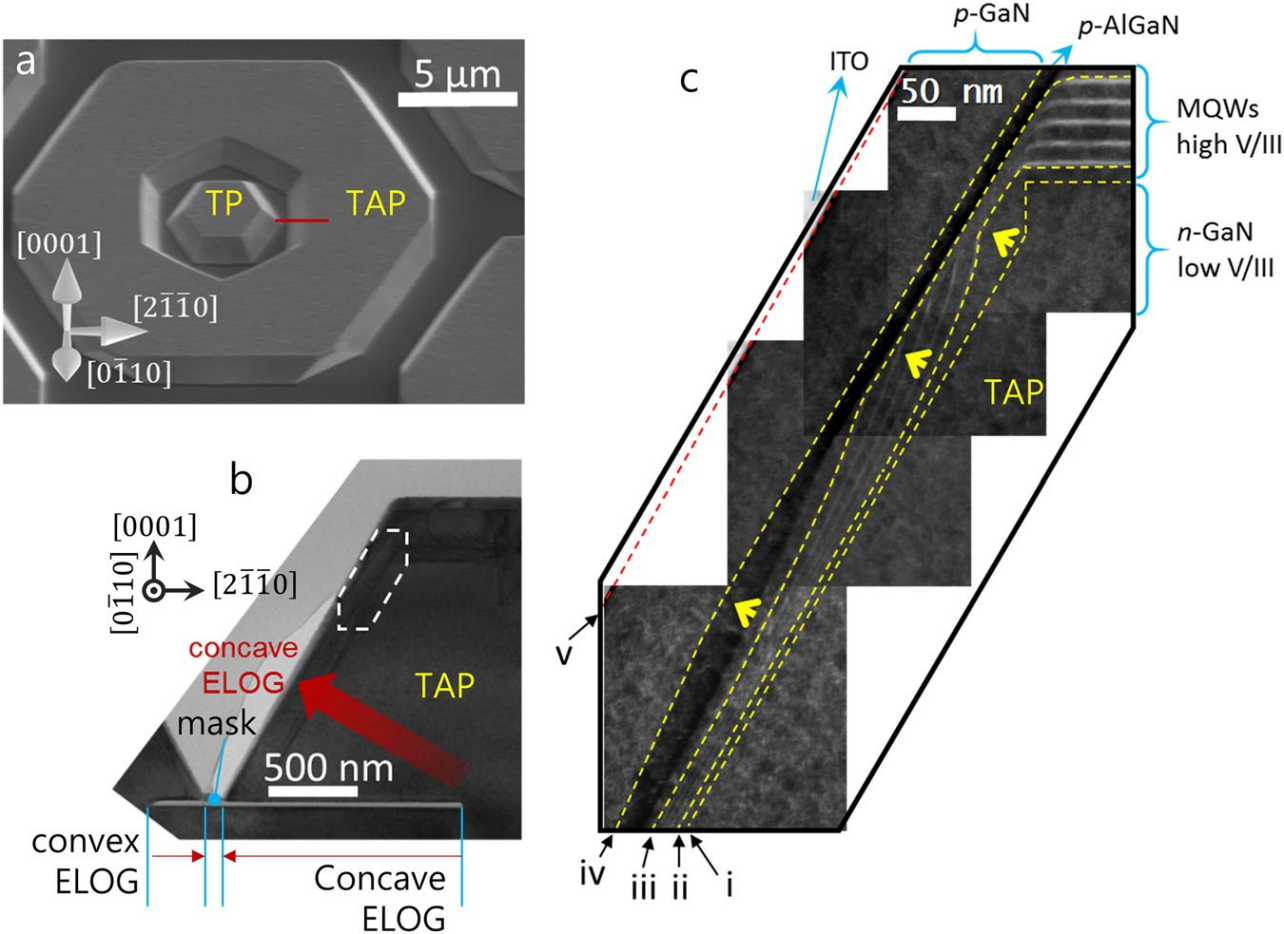
(**a**) A top-down SEM image of sample B. (**b**) A bright field TEM image of the inner TAP structure along the < 1$\bar{1}$10 > axis. (**c**) High magnification HAADF-STEM image of inner TAP structure with full LED structures.

Figure 4(a) shows a bird’s eye view of an SEM image of the double concentric TP and TAP 3D structure. A transmission electron microscopy (TEM) specimen was fabricated by FIB milling along the indicated red line. As shown in Fig. 4(b), an overview of the TEM specimen was taken by a bright field TEM image, where the SiN_*x*_ dielectric mask is clearly observed. Finally, we took a high-angle annular dark field scanning TEM (HAADF-STEM) image (Fig. 4(c)) of the upper part of the TAP structure indicated by the white dashed box in Fig. 4(b). Since an interface between layers directly indicates the shape of the growth front formed at the same moment, the time evolution of the growth front can be deduced from the HADDF-STEM image. In Fig. 4(c), the five dashed guidelines, (i) to (v), indicate the interface lines between: (i) the *n*-GaN 3D structure and the first barrier; (ii) the first barrier and first well; (iii) the 5^th^ well and 6^th^ barrier; (iv) the *p*-AlGaN and *p*-GaN layers; and (v) the *p*-GaN and ITO layers, respectively. As we have already discussed, in Fig. 3(c,d), the *a*-plane {$11\bar{2}0$} is formed above the semi-polar {$11\bar{2}2$} facets on the interior of the TAP structures under low V/III ratio conditions. However, above the aftergrown *n*-GaN layer (i.e., guide line (i)), the V/III ratio increases by over a factor of 10, and the environment becomes nitrogen-rich. Therefore, according the kinetic Wulff plot, the N-polarity of semi-polar {$11\bar{2}2$} facets is more stable than the non-polar of *a*-plane {$11\bar{2}0$} facets^11^. In Fig. 4(c), we found that growth rate increases locally from top to bottom (indicated by the yellow arrows) after the V/III ratio changes. In order to transform the growth front of the *a*-plane {$11\bar{2}0$} facets to the semi-polar {$11\bar{2}2$} facets, a growth front of {$11\bar{2}$ *x*} facets (where 0 < *x* < 2) is temporarily created as time proceeds. This interesting phenomenon can also be theoretically explained with reference to Q. Sun *et al*.^24^. According to their 3D *v*-plot, the growth rate of *a*-plane {$11\bar{2}0$} facets is dramatically increased by increasing the V/III ratio. Using this growth front transformation, a large energy gap gradient can be generated that depends on the growth facets and their location, and leads to variations in MQW thickness, strain, and In composition. Using the *a*-plane facets in 3D structure we addressed, color rendering index of 3D structure LEDs^13^ can be intentionally controlled. In addition, photovoltaic cells and nonlinear photonic diode^25^ can be precisely designed.

## Conclusions

Arch-shaped *a*-plane {$11\bar{2}0$} facets can be fabricated on the exterior of a TAP structure, and above semi-polar facets through the use of a particular geometrical design of dielectric mask, and by adjustment of the growth time. The area covered by growth facets in an SAG 3D structure may also be controlled. As our previous results showed^13^, these additional facets offer a wide range of useful features for applications, such as a high color rendering index for LEDs, and a broad absorption band for photovoltaic applications. Both of these structural properties may be taken advantage of in applications since multiple, spatially separated growth facets can emit or absorb various wavelengths of light. Furthermore, by changing the SAG growth parameters, we found a growth front transformation of {$11\bar{2}$ *x*} facets (where 0 < *x* < 2), resulting in variations of the indium composition, as well as the quantum well thickness during active layer growth. This phenomenon can be adopted to fabricate broadband LEDs, photovoltaic cells, or nonlinear photonic diodes.

## Methods

### MOCVD Growth

A 30-nm-thick SiN_*x*_ layer was deposited on a 3-μm-thick GaN template grown on a *c*-plane sapphire substrate. Double concentric truncated pyramid structures of GaN were grown by MOCVD through the SAG technique, using a pair of concentric circular and ring patterns. Five pairs of InGaN/GaN MQWs, *p*-AlGaN, *p*-GaN layers were grown on the double concentric truncated pyramid structure of *n*-GaN.

### Structural characterization

Ion beam milling was used to produce the cross-sectional TEM specimen, and the cross-sectional SEM images were taken using a dual beam focused ion beam (Helios 600 NanoLab, FEI, USA). Bright field TEM and HAADF-STEM images were taken using a double Cs-corrected TEM (JEM-ARM200F JEOL, Japan).

## Electronic supplementary material

Supplementary information
