# Supplementary material for: Formation of a-plane facets in three-dimensional hexagonal GaN structures for photonic devices
Source: Sci Rep. 2017 Aug 24;7:9356. doi: 10.1038/s41598-017-09782-1 (PMC5571163; doi:10.1038/s41598-017-09782-1)
Supplement: Supplementary file 1 — Supplementary information [file 41598_2017_9782_MOESM1_ESM.doc]

**Formation of *a*-plane facets in three-dimensional hexagonal GaN structures for photonic devices**

**Seung-Hyuk Lim,**^†^ **Young Chul Sim, Yang-Seok Yoo, Sunghan Choi, Sangwon Lee, and Yong-Hoon Cho***

Department of Physics, Korea Advanced Institute of Science and Technology, Daejeon 34141, Republic of Korea.

†Current address: Seung-Hyuk Lim

Department of Physics, Chemistry, and Biology (IFM), Semiconductor Materials, Linköping University, SE-58183 Linköping, Sweden

*Correspondence: Prof. Yong-Hoon Cho

Department of Physics, Korea Advanced Institute of Science and Technology, Daejeon 34141, Republic of Korea. Tel: (82) 42-350-2549, Email: [yhc@kaist.ac.kr](mailto:yhc@kaist.ac.kr)

**Deducing the lateral growth distance**

Since the focused ion beam cutting line is along the [21¯1¯0] direction, the growth distance, d_es_, from the circular opening to the edge was measured along the {101¯1} direction (see Figure S1). In order to compare the growth rate between convex and concave lateral overgrown regions, the growth distance along {101¯1} (d_s_) is deduced with simple trigonometric methods as follows:


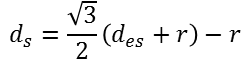
 (S1)

where *r* is radius of circular opening.


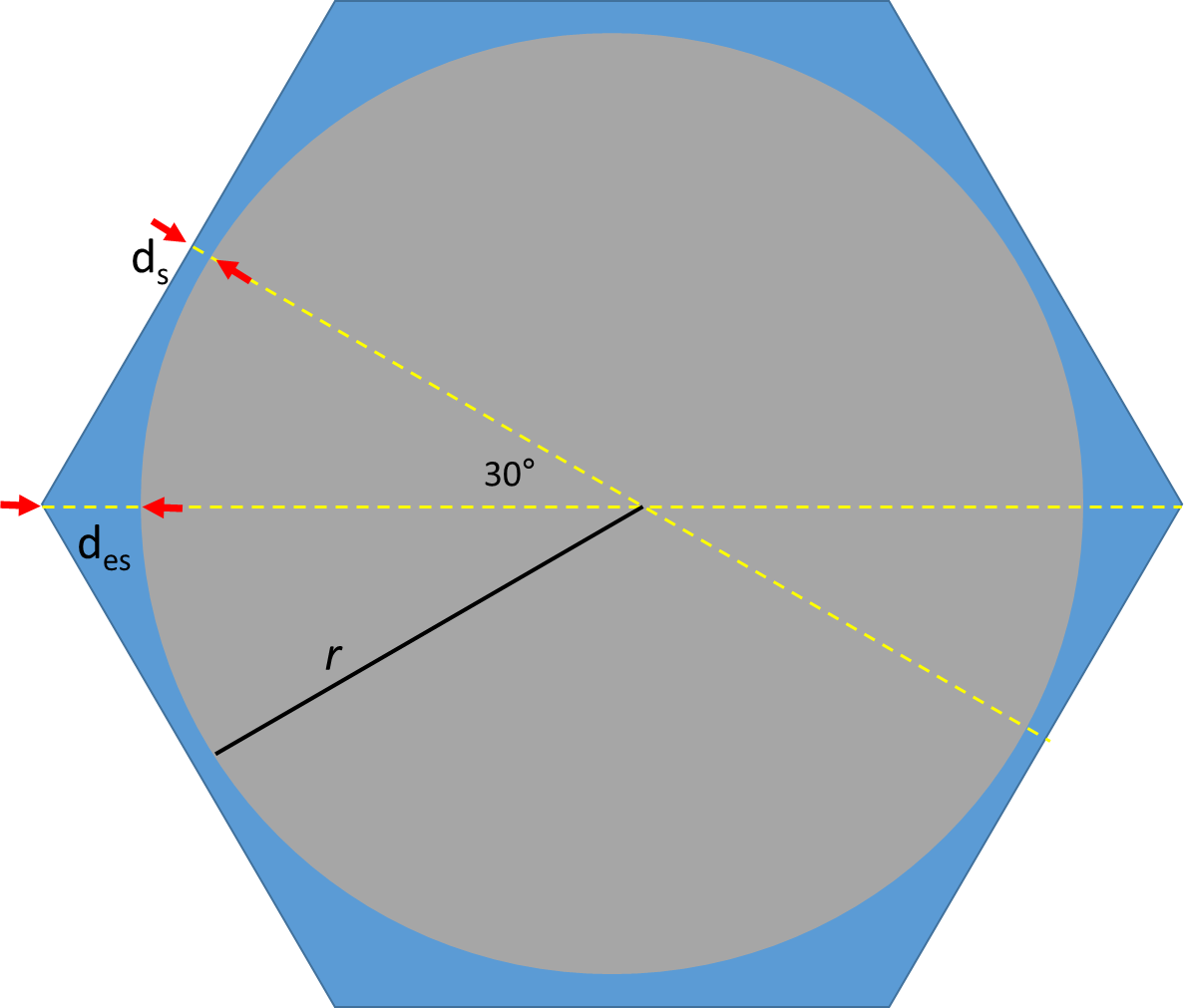


**Figure S1| Top view schematic used to deduce the lateral growth distance.** Red arrows indicate d_es_ and d_s_.
